# Changes in brain arousal (EEG-vigilance) after therapeutic sleep deprivation in depressive patients and healthy controls

**DOI:** 10.1038/s41598-018-33228-x

**Published:** 2018-10-10

**Authors:** Christian Sander, Jonathan M. Schmidt, Roland Mergl, Frank M. Schmidt, Ulrich Hegerl

**Affiliations:** 1 Department of Psychiatry and Psychotherapy, University Hospital Leipzig, Leipzig, Germany; 2 Medical Faculty, University of Leipzig, Leipzig, Germany

## Abstract

Depressed patients frequently exhibit a hyperstable brain arousal regulation. According to the arousal regulation model of affective disorders, the antidepressant effect of therapeutic sleep deprivation could be achieved by counter-acting this dysregulation. We investigated the impact of partial sleep deprivation (PSD) on EEG-vigilance (an indicator of brain arousal regulation) in depressed patients (n = 27) and healthy controls (n = 16). PSD was hypothesized to cause a more prominent destabilisation of brain arousal regulation in depressed patients (reflected by increased occurrence of lower EEG-vigilance stages). Furthermore, it was studied whether responders (n = 17) exhibit a more stable baseline brain arousal regulation and would show a more prominent arousal destabilisation after PSD than non-responders (n = 10). Before PSD, patients showed a more stable EEG-vigilance with less declines to lower vigilance stages compared to controls. Contrary to the hypothesis, a greater destabilisation of brain arousal after PSD was seen in controls. Within the patient sample, responders generally showed a less stable EEG-vigilance, especially after PSD when we found significant differences compared to non-responders. EEG-vigilance in non-responders showed only little change from baseline to after PSD. In summary, PSD had a destabilizing impact on brain arousal regulation in healthy controls whereas depressed patients reacted heterogeneously depending on the outcome of treatment.

## Introduction

### Sleep deprivation

About 60% of depressed patients show considerable improvement or even remission after therapeutic sleep deprivation^1^. With its immediate effect, sleep deprivation has an important advantage over other conventional treatments with longer latencies^2^. Partial sleep deprivation, where patients have to stay awake in the second half of the night, is reported to have a similar effect as total sleep deprivation but is better tolerated by patients^3^. The majority of patients experience a relapse of depressive symptoms after sleep in the following night, identified as recovery night. It seems essential not to nap during the day after sleep deprivation as this can trigger a relapse of depressive symptoms^4^. Several theories have been endeavouring to explain the antidepressant effects of sleep deprivation therapy^1,5–9^. For example, a generalised depressiogenic effect of sleep due to specific substances released during sleep was suspected and a link to cortisol was suggested as this was found to be increased in depressed patients^1^. Other theories suggest that sleep deprivation leads to a correction of disturbed diurnal rhythms, as depressed patients often show deviations in sleep, temperature and hormonal secretion^5,7^. Up to now, the biological mechanisms underlying the therapeutic effect of sleep deprivation have not been fully understood. A further explanation can be deduced from the arousal regulation model of affective disorders^10^.

### Brain arousal and EEG-vigilance regulation

The Research Domain Criteria project^11^ defines arousal as a relevant dimension for the investigation of mental disorders. Brain arousal regulation, as a key aspect, can best be assessed using electroencephalography (EEG) and – besides the well-known sleep stages – distinctive states can be distinguished during the transition wakefulness to sleep onset (called EEG-vigilance stages, see methods section). Stage 0 represents the activated state of mind as seen in mental effort; Stage A (with sub-stages A1, A2 and A3) represents relaxed wakefulness; Stage B (with sub-stages B1, B2/3) corresponds to drowsiness, and Stage C represents sleep onset. There are several studies on changes of EEG activity during the transition from active wakefulness to sleep onset that endorse this classification^12–19^.

Patients with Major Depression have been found to exhibit a tonically high brain arousal level with less and delayed declines to lower EEG-vigilance stages compared to health controls^20^. Brain arousal regulation can be examined by studying the temporal sequence of EEG-vigilance stages recorded over a prolonged period of time. Considerable inter-individual differences have been shown^10,21^ and three prototypical arousal regulation patterns can be described: Most people show a gradual decline from higher to lower vigilance stages (adaptive arousal regulation). In other individuals the decline to lower EEG-vigilance stages happens immediately after eye closure (instable arousal regulation), while a third group remains in rather high vigilance stages during the whole recording period (hyperstable arousal regulation).

The disturbed regulation of brain arousal has a pathogenetic relevance in the arousal regulation model of affective disorders^10^. An instable arousal regulation has been seen in patients suffering from mania or ADHD, whereas in depressed patients the hyperstable arousal regulation has consistently been shown by our group^21–23^. This is in accordance to the high inner tension reported by depressed patients and the sleep alterations typically found in depression^24,25^. On the behavioural level, many depressed patients exhibit avoidance and social withdrawal. This observation is hypothesized to reflect an auto-regulatory response to their hyperstable arousal regulation. Within this explanatory model, therapeutic sleep deprivation is supposed to counteract the arousal dysregulation by strengthening sleep promoting brain mechanisms. An increase in homeostatic sleep pressure due to sleep deprivation could antagonize the hyperstable brain arousal regulation, thus leading to an improvement of mood. The deterioration of mood after sleep deprivation observed in non-depressed subjects^26,27^ implies that a destabilisation of brain arousal would have detrimental effects if no hyperstable brain arousal regulation was present.

The aim of this study is to test hypotheses resulting from the arousal regulation model. We compared the EEG-vigilance (as an indicator for brain arousal regulation) in depressed patients and healthy controls before and after sleep deprivation, hypothesizing that sleep deprivation generally causes a destabilisation of EEG-vigilance, especially in the depressed patients. Within the patient sample, we expected the responders to sleep deprivation to exhibit a higher level and more stable regulation of EEG-vigilance at baseline than non-responders and to show a larger destabilisation of EEG-vigilance after sleep deprivation. This was based on the idea that those patients showing a more hyperstable brain regulation have more potential for modification through the intervention.

## Methods and Materials

### Subjects

Depressed in-patients of the Department of Psychiatry and Psychotherapy at the University Hospital of Leipzig are treated according to a fixed treatment algorithm which includes partial sleep deprivation (PSD) as an additional intervention to pharmacological treatment and psychotherapy. We recruited patients undergoing PSD consecutively between 2011 and 2014. Inclusion criteria were:

- a current depressive episode (ICD-10-criteria: F32 and F33) and antidepressant treatment,
- at least 8 points in the 17-item Hamilton Depression Rating Scale^28^,
- a minimum age of 18 and
- written informed consent to participate in the study and capacity to consent.

Exclusion criteria were

- psychiatric co-morbidities according to ICD-10-criteria (F0x.x, F1x.x, F2x.x),
- psychotic symptoms,
- severe neurological or cardiovascular disease,
- drug addiction or alcohol addiction in the past or abuse in the last six months,
- electroconvulsive therapy in the last six months and
- pregnancy or breastfeeding.

All patients except one were on antidepressant medication during the study (N = 14 SSRI monotherapy, N = 7 SSRI + Mirtazapin, N = 3 Mirtazapin monotherapy, N = 2 other medication, N = 1 no medication).

Healthy controls were recruited by public announcement. They had to be 18 years or older and give written consent. Exclusion criteria were all those valid for depressive patients (a–f) plus (g) prevalence of a psychiatric disease according to ICD-10-criteria (F0x.x-F5x.x and F60.3) and (h) shift work at the time of the investigation.

In total, 40 patients and 20 healthy controls were recruited for the study. All study assessments were performed in the course of two consecutive study days: Day 1 (baseline), the day before the sleep deprivation night, and Day 2, the day after the sleep deprivation night. Of the recruited patients, 4 patients withdrew from the study after sleep deprivation, therefore not participating in the EEG assessment on day 2. Five patients and two controls were excluded since at least one EEG could not be analysed according to VIGALL requirements. Finally, 4 patients and 2 controls had to be excluded due to non-adherence to the sleep deprivation protocol hinted by actigraphy (see section 2.2). Therefore, a total of 27 patients and 16 controls were included into the final analyses. Patients and controls were comparable concerning age and sex distribution but obviously differed significantly in respect to depressive symptomatology (see Table 1, left part).

**Table 1:** Baseline-characteristics of patients versus healthy controls (left) and of depressed patients responding versus non-responding to partial sleep deprivation (right).

	Patients (N = 27)	Controls (N = 16)		Responder (N = 17)	Non-Responder (N = 10)	
Age (years)	40.33 (±13.981)	37.94 (±13.309)	t = 0.553 p = 0.583	35.71 (±10.457)	48.20 (±16.158)	t = −2.448 p = 0.022
Sex (m/f)	10/17	7/9	Χ^2^ = 0.189 p = 0.663	5/12	5/5	X^2^ = 1.144 p = 0.285
HDRS-17	15.63 (±3.835)	1.25 (±1.949)	t = 16.260 p < 0.001	15.94 (±3.473)	15.10 (±4.533)	t = 0.543 p = 0.592
IDS-C	31.48 (±8.196)	1.85 (±2.734)	t = 16.933 p < 0.001	32.94 (±7.058)	29.00 (±9.730)	t = 1.218 p = 0.235

Shown are means (±standard Deviations).

Annotations: HDRS-17 = Hamilton Depression Rating Scale (17 item version); IDS-C = Inventory of Depressive Symptomatology (Clinician Rated).

### Procedures

#### Sleep deprivation

All participants underwent partial sleep deprivation (PSD). On the day before PSD, patients were asked to go to bed at around 9 pm. At 1am they were awoken by the nursing staff and spent the rest of the night in a group room under supervision by the nursing staff. Patients were asked to stay awake until around 8 pm and were then allowed to go to bed for recovery sleep. Healthy controls were supposed to undergo similar procedures, however in their own homes. They were also requested to get up at 1am in the PSD night and were allowed to spend the rest of the night with activities of their own choosing. All study assessments were carried out in the hospital. To ensure adherence to the study protocol, all participants were requested to wear an actigraphy device (Actiwatch Spectrum, Phillips Respironics) during the study. Actigraphy data was screened visually for signs of non-adherence to the study requirements.

#### EEG acquisition

To assess the influence of sleep deprivation on vigilance regulation, a 15-min resting EEG with closed eyes was recorded on both days using a 40-channel QuickAmp amplifier (Brain Products GmbH, Gilching Germany). Sampling rate was 1000 Hz; impedances were kept below 10 kΩ. Thirty-one electrodes were positioned via ElectroCaps (Brain Products, Germany) according to the international 10–20 system. Participants were seated in a comfortable chair in a sound-attenuated room and were asked to keep their eyes closed. They were instructed to rest and try neither to stay awake nor to fall asleep on purpose. All EEG-examinations took place between 12 pm and 3 pm.

#### Questionnaires

Depression severity was rated on the Hamilton Depression Rating Scale (HDRS)^28^. At baseline, symptoms were assessed using a combination of the HDRS and the Inventory of Depressive Symptomatology, Clinician Rating (IDS-C)^29^. Information was derived from a semi-standardized interview^30^, which was performed either preceding the EEG-recordings or afterwards. Due to the daily assessment and the aim of assessing changes in symptoms due to sleep deprivation, the HDRS-score according to Bech *et al*. (HAMD_Bech_) was calculated^31^, which comprises only 6 change sensitive items (Depressed mood, feelings of guilt, work and activities, retardation, anxiety, somatic symptoms). Response to sleep deprivation was defined as a score reduction by more than 50% from baseline in the HDRS_Bech_.

Mood was rated by the participants twice a day (in the morning (around 7:30 am) and at the time of the EEG recording) using the “Aktuelle Stimmungsskala” (ASTS)^32^, a German shortened form of the Profile of Mood States (POMS)^33^ which includes 19 mood descriptions to be rated on a 7 point Likert scale. Five scores can be calculated attesting to the current level of sadness, hopelessness, tiredness, anger and positive mood. Furthermore, the level of wakefulness before the EEG recording was assessed using the Stanford Sleepiness Scale (SSS)^34^. Questionnaires with missing information were not included in the explorative analyses of changes in subjective mood.

### EEG-vigilance classification

EEG pre-processing was done with the software package BrainVision Analyzer 2 (Brain Products GmbH, Germany) according to the procedures described elsewhere^35^. Afterwards, vigilance classification was performed using VIGALL 2.1, a semi-automatic computer-algorithm that allocates an EEG-vigilance stage to every 1-second EEG segment in a continuous resting EEG (for detailed descriptions of the scoring algorithm see^36^):

- Stage 0 represents the activated state of mind as seen in mental effort. In stage 0 a low amplitude EEG with non-alpha-activity is seen, typically without slow horizontal eye-movements.
- Stage A represents relaxed wakefulness: The EEG shows dominant alpha activity. Stage A can be subdivided into A1, A2 and A3, using the degree of shifting of alpha activity from occipital to anterior brain regions. Stage A1 is characterised by predominant occipital alpha-activity. In A2/3 stages, alpha-activity shifts to frontal and temporal regions and the amplitude decreases.
- Stage B corresponds to drowsiness and can be divided into sub-stages B1 (low amplitude EEG without alpha-activity, paralleled by slow horizontal eye movements) and B2/3 (dominated by theta and/or delta activity).
- Stage C reflects sleep onset and is characterised by sleep spindles and K-complexes.

Results of the VIGALL classification were imported into a customized Excel template with Visual Basic for Applications (VBA) macros (Microsoft) to compute several vigilance parameters:

- The absolute amount of vigilance stages (0, A1, A2/3, B1, B2/3 and C) as well as percentage (amount * 100/total number of non-artefact segments) over the whole recoding period as well as for each recording minute.
- Each vigilance stage was assigned a numerical score ranging from 1 (lowest stage C) to 6 (highest stage 0). Mean vigilance values (MVV) were calculated by averaging the scores of all non-artefact segments over the whole recording period as well as for each recording minute.
- An arousal stability score (ASS) was determined for each vigilance time course to quantify the speed and extend of the respective vigilance decline. Successive blocks of 1 min duration were analyzed concerning fulfilment of one of the following criteria: (I) at least 2/3 of all segments classified as 0/A or 0/A1 stages; (II) 1/3 of all segments classified as B stages (B1 + B2/3); (III) at least 1/3 of segments classified as B2/3 stages; (IV) occurrence of at least 1 C stage. If within the whole recording only criterion I was fulfilled, a high ASS of 11 or 10 was given corresponding to the high amount of arousal stability. If one of the other criteria was fulfilled, the respective ASS score (9 to 1) was given as seen in Table 2.

**Table 2:** Arousal Stability Score corresponding to occurrence of certain vigilance stages during a 15-min resting EEG.

Score	Stability Level (criterion)	Operational definition
11	Level 1: less than 1/3 of all segments not classified as 0/A- or 0/A1-stages	rigidity, only appearance of 0 and A1 stages
10		rigidity, only appearance of 0 and A stages
9	Level 2: at least 1/3 of all segments classified as B (B1 + B2/3)-stages	stage B emerged in minute 11–15
8		stage B emerged in minute 6–10
7		stage B emerged in minute 1–5
6	Level 3: at least 1/3 of segments classified as B2/3-stages	stage B2/3 emerged in minute 11–15
5		stage B2/3 emerged in minute 6–10
4		stage B2/3 emerged in minute 1–5
3	Level 4: occurrence of at least 1 C-stage	stage C emerged in minute 11–15
2		stage C emerged in minute 6–10
1		stage C emerged in minute 1–5

### Statistics

Data analysis was carried out using the SPSS 18 data analysis package. Differences between patients and controls as well as responders and non-responders concerning questionnaire results and clinical data were performed using parametric or non-parametric tests according to data level.

To investigate the impact of sleep deprivation on Mean Vigilance Values (MVV) we performed repeated measures ANOVAs with ‘group’ (patients, controls) as between subject factor and ‘day’ (baseline, after PSD) and ‘recording time’ (minute 1 to 15) as within-subject factors. Due to the ordinal character of the Arousal Stability Score (ASS), we performed a generalized estimating equation (GEE) with ‘group’ and ‘day’ as factors and ASS as dependent variable. Since responders and non-responders were found to differ in age, repeated measure ANCOVAs or GEE, respectively, were performed for the responder vs. non-responder comparisons, with ‘status’ (responders, non-responders) as between subject and ‘day’ as within-subject factor; ‘age’ was included as covariate (main effects of age and age * day as well as age * time interactions were included in the model).

We used a Greenhous-Geisser correction in case of significant findings in Mauchly’s sphericity test. To correct for multiple testing we used an alpha-adjusted significance level of p = 0.025 in view of two outcome parameters (MVV, ASS). For post-hoc tests, independent and dependent t-tests or Mann-Whitney-U test, Wilcoxon test and Friedman Test were used according to the respective data type. Additionally, the above mentioned repeated measures ANOVAs/ANCOVAs were performed separately for four EEG-vigilance stage combinations (0/A1 vs. A2/3 vs. B1 vs. B2/3 + C) as well as questionnaire scores with ‘group’ or ‘status’ as between subject factors and ‘day’ and ‘recording time’ as within-subject factors.

### Ethical considerations

The present study was approved by the local ethics committee of the University of Leipzig (#327-10-08112010 & #308-12-24092012). Written informed consent was obtained from all participants and all procedures were performed in accordance with relevant guidelines and regulations.

## Results

### Depressed Patients vs. Health Controls

#### Vigilance Classification Results

**Figure 1:**
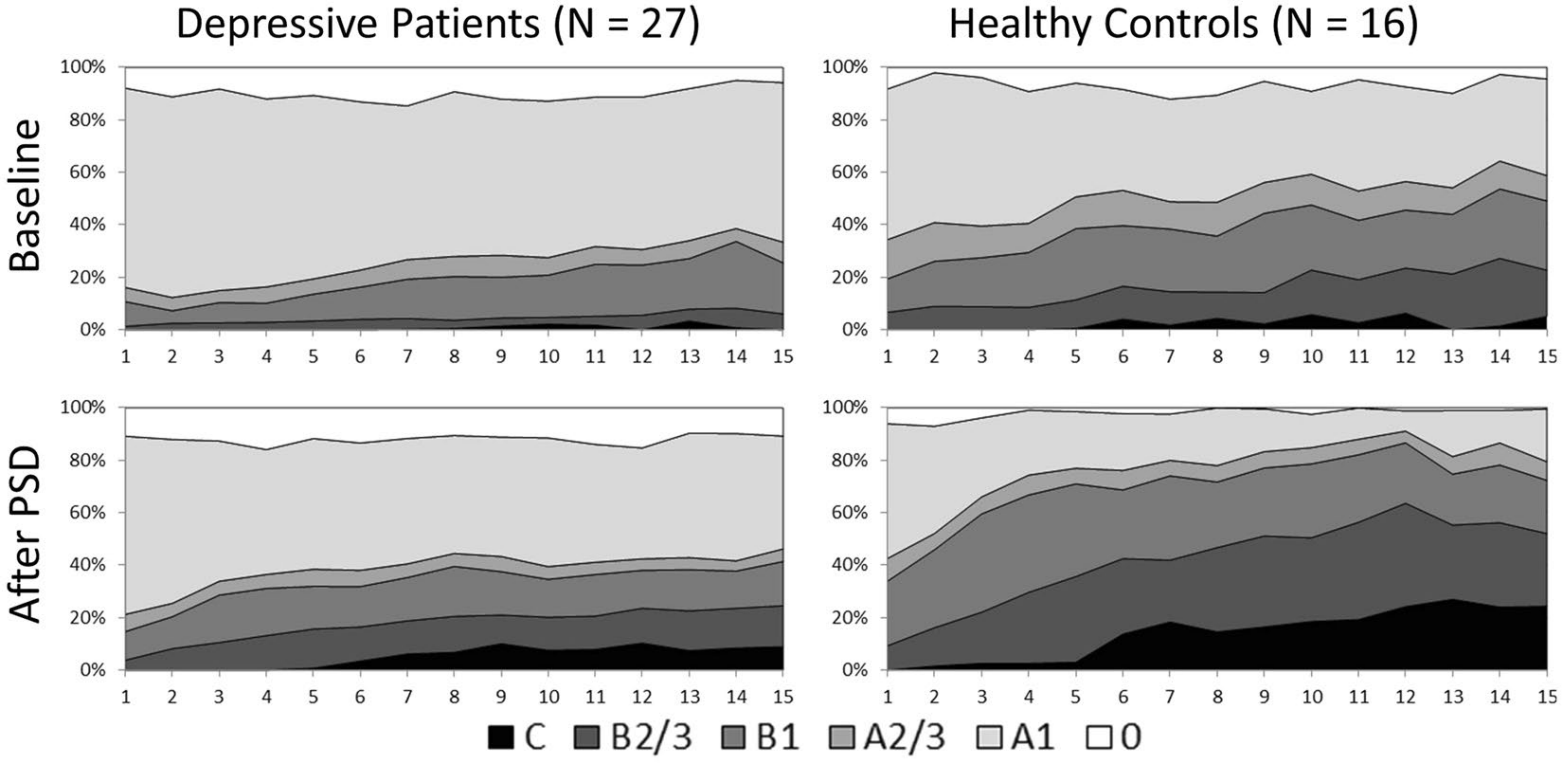
Time-course of EEG-vigilance stages during 15 minutes of resting EEG between patients and healthy controls on day 1 (baseline, upper row) and day 2 (after sleep deprivation (PSD), lower row).

Patients and controls showed significant differences concerning the time-course of EEG-vigilance stages during the 15 min EEG recordings on the two assessment days (see Fig. 1). Patients remained in higher vigilance stages for longer over the recording period on both assessments, whereas controls reached lower vigilance stages more frequently. This was most pronounced after sleep deprivation when the EEGs of controls were dominated by lower vigilance stages whereas a much lesser increase in lower EEG vigilance stages was noticeable in the depressive sample. Accordingly, rmANOVA results for the mean vigilance values (MVV) (see Table 3, left part) revealed significant main effects of group, recording time, and day (all p < 0.001) and a time * day interaction (p = 0.004).

**Table 3:** Results on changes of the Mean Vigilance Value and the Arousal Stability Score in patients vs. healthy controls (left part) and responders vs. non-responders to sleep deprivation (right part) from baseline to after sleep deprivation.

Patients vs. Controls		Responder vs. Non-Responder	
Mean Vigilance Value^A^		Mean Vigilance Value^B^	
ME group	**F = 16**.**580; p < 0**.**001**	ME status	**F = 10**.**742; p = 0**.**003**
ME time	**F = 19**.**325; p < 0**.**001**	ME time	F = 1.028; p = 0.425
ME day	**F = 25**.**164; p < 0**.**001**	ME day	F = 0.123; p = 0.729
Group * time	F = 2.333; p = 0.059	Status * time	F = 2.594; p = 0.044
Group * day	F = 3.898; p = 0.055	Status * day	F = 2.944; p = 0.099
Time * day	**F = 3**.**434; p = 0**.**004**	Time * day	F = 1.236; p = 0.296
Group * time * day	F = 1.278; p = 0.271	Status * time * day	F = 0.759; p = 0.582
**Arousal Stability Score** ^**C**^		**Arousal Stability Score** ^**D**^	
ME group	**X²** = **14**.**104; p** < **0**.**001**	ME status	**X**² = **13**.**877; p < 0**.**000**
ME day	**X²** = **10**.**627; p = 0**.**001**	ME day	X² = 1.338; p = 0.247
Group * day	X² = 2.938; p = 0.087	Status * day	X² = 0.498; p = 0.397

Annotations: ME = Main Effect; Group (depressive patients vs. healthy controls), Status (responders vs. non-responders), time (recording minutes 1–15), and day (assessment days: baseline vs. after sleep deprivation).

^A^Repeated measure ANOVA.

^B^Repeated measures ANCOVA (with age as covariate).

^C^Generalized estimating equation.

^D^Generalized estimating equation, including age as cavariate (main effects of age and age * day as well as age * time interactions were considered).

**Figure 2:**
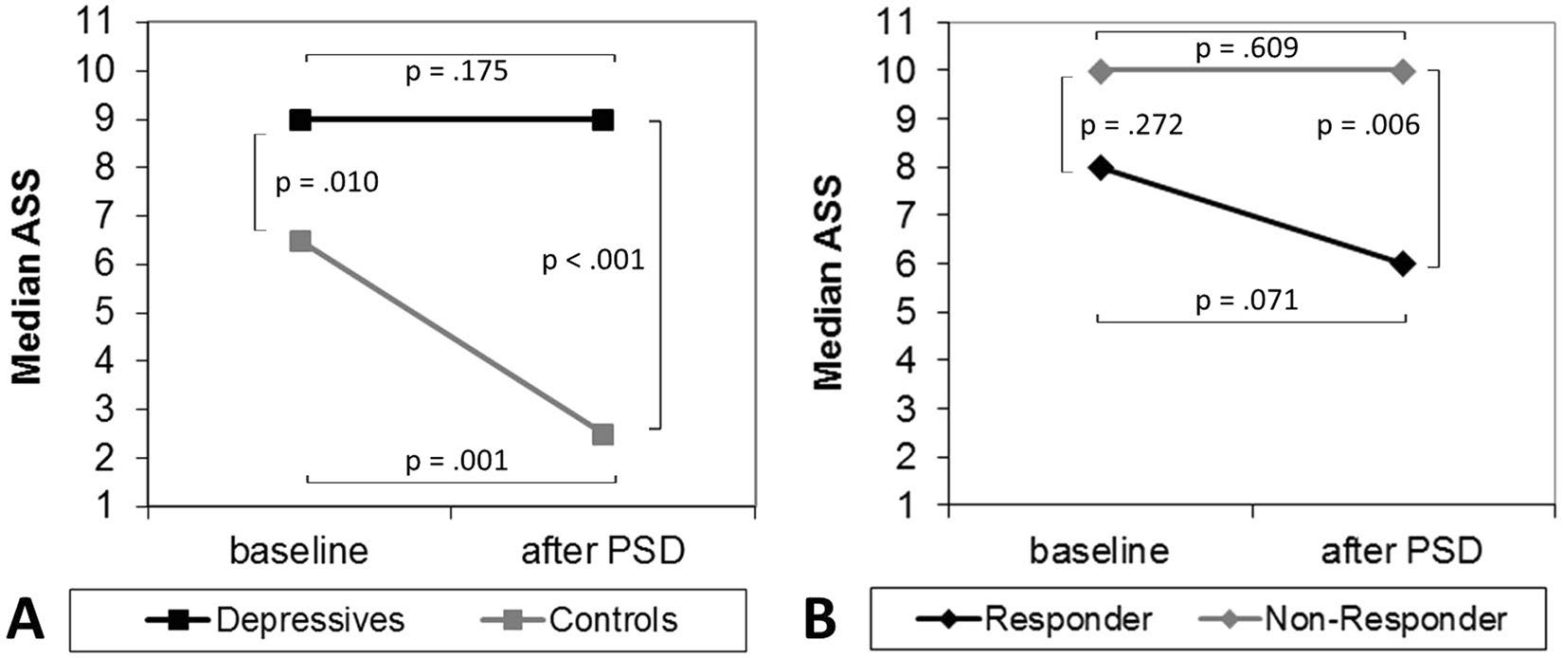
Comparison of Median Arousal Stability Scores (ASS) on day 1 (baseline) and day 2 (after sleep deprivation, PSD) in patients versus controls (part **A**) as well as responders versus non-responders (part **B**).

Sleep deprivation also had a different impact on the Arousal Stability Scores (ASS) of patients and controls (see Fig. 2, part A). A generalized estimating equation (GEE, see Table 3, left part) revealed a main effect of group (X² = 14.104; p < 0.001) and day (X² = 10.627; p = 0.001). Post hoc tests showed that patients had a significantly higher ASS than controls on both assessment days (baseline: Z = −2.560, p = 0.010; after PSD: Z = −3.510, p < 0.001) and did not show significant changes after PSD (Z = −1.357, p = 0.175). In the control sample, the PSD resulted in significant ASS changes (Z = −3.262, p = 0.001).

Separate post-hoc rmANOVAs for EEG-vigilance stage combinations revealed significant differences between groups and between assessment days for stages 0/A1 and B2/3 + C (see Supplement Table S1).

#### Mood and sleepiness ratings

Mood ratings at baseline and after sleep deprivation are shown in Table 4. In patients, sleep deprivation had a clear effect on HDRS_Bech_-scores as baseline scores were significantly higher than those after PSD (paired t = 7.353, p < 0.001). Similar effects were seen concerning ASTS ratings on sadness and hopelessness and the reverse effect was found concerning positive mood (see Supplement Table S2).

**Table 4:** Mood and sleepiness ratings (Means (±Standard Deviations)) in patients vs. healthy controls (left part) and responding vs. non-responding patients (right part) at baseline and after partial sleep deprivation (PSD).

		Patients vs. Controls			
		N	mean (s.d.)	mean (s.d.)	Post-hoc Tests
Depression Severity (HDRS_Bech_)	Baseline	27/16	7.96 (±2.244)	0.50 (±0.816)	t = 15.622; p < 0.001
	After PSD	27/0	3.48 (±2.44)	—	—
Sleepiness (SSS) before EEG	Baseline	26/16	3.73 (±1.251)	1.69 (±0.479)	t = 7.486; p < 0.001
	After PSD	27/16	3.56 (±1.649)	2.69 (±1.250)	t = 1.816; p = 0.077
**Responder vs. Non-Responder**					
Depression Severity (HDRS_Bech_)	Baseline	17/10	8.41 (±2.033)	7.20 (±2.486)	t = 1.378; p = 0.180
	After PSD	17/10	2.06 (±1.435)	5.90 (±2.644)	t = −4.923; p < 0.001
Sleepiness (SSS) before EEG	Baseline	16/10	3.88 (±1.204)	3.50 (±1.354)	t = 0.737; p = 0.468
	After PSD	17/10	3.18 (±1.667)	4.20 (±1.476)	t = −1.604; p = 0.121

Annotations: HDRS_Bech_ = Hamilton Depression Rating Scale Score according to Bech *et al*.^22^; SSS = Stanford Sleepiness Scale; s.d. = standard deviation.

Contrary to the mood ratings, PSD had a higher impact on sleepiness ratings (SSS) in controls than in patients. There was a distinct main effect of group (F = 21.121, p < 0.001) and a significant group * day interaction (F = 5.114, p = 0.029). Sleepiness ratings remained unchanged in patients (paired t = 0.231, p = 0.819) but there was a significant increase in reported sleepiness in the control group (t = −3.651, p = 0.002). A similar effect was seen in the ASTS ratings on tiredness, where no significant group difference was found after PSD (see Supplement Table S2).

### Responder vs. Non-Responder

To investigate whether assessment of EEG-vigilance regulation could be used as a response predictor for sleep deprivation treatment, patients were divided into two status groups according to changes in their HDRS_Bech_-score from baseline to after PSD (response was defined as reduction by more than 50% from baseline): Of the 27 patients, 17 (63.0%) were classified as responders, 10 as non-responders (37.0%). Table 1 (right part) shows the characteristics of responders and non-responders. At baseline, no significant difference between the two groups could be seen in terms of clinician-rated depression severity (IDS-C). However, responders were significantly younger than non-responders.

#### Vigilance Classification Results

**Figure 3:**
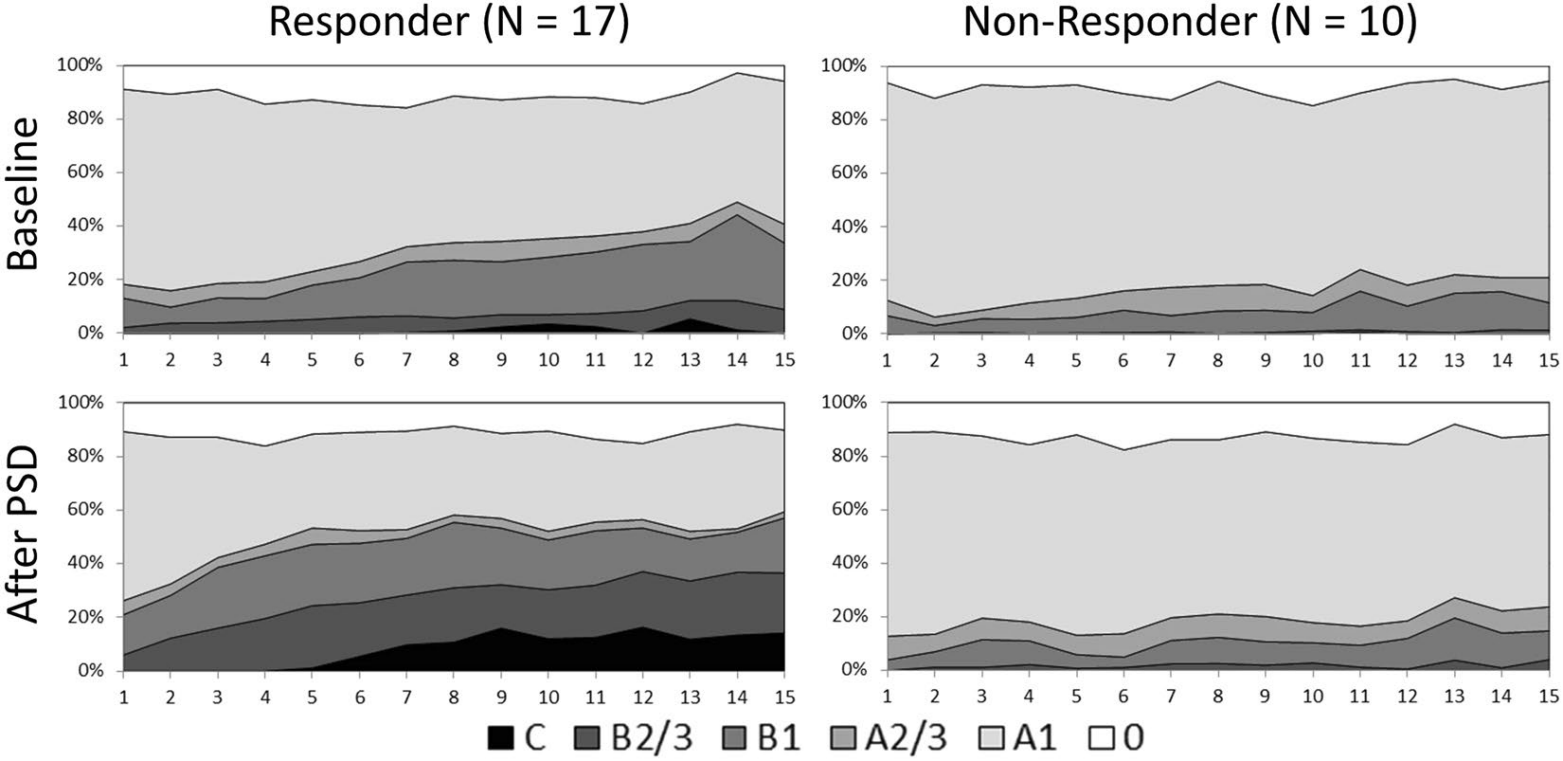
Time-course of EEG-vigilance stages during 15 minutes of resting EEG in responders and non-responders to sleep deprivation on day 1 (baseline, upper row) and day 2 (after sleep deprivation (PSD), lower row).

The time-course of EEG-vigilance stages during the two assessments (baseline, after PSD) is shown in Fig. 3 for both status groups. Overall, compared to non-responders responders reached lower vigilance stages more often during the course of the resting EEG, especially after PSD. Accordingly, age-adjusted rmANCOVA results on the MVV (see Table 3, right part) showed significant main effects of status (p = 0.003). A post-hoc ANCOVA comprising only baseline data, revealed a significant main effect of status (F = 6.309; p = 0.019), with responders exhibiting lower MVV scores than non-responders.

When status groups were compared concerning their Arousal Stability Scores (ASS), we generally found higher scores in non-responders, indicating a more stable brain arousal regulation (see Fig. 2, part B). The age-adjusted GEE analysis accordingly resulted in a significant main effect of status (X² = 13.877; p < 0.001). Post-hoc tests showed no significant difference between responders and non-responders at baseline (Z = −1.097, p = 0.272) but after PSD (Z = −2.769, p = 0.006).

In separate explorative rmANCOVAs for EEG-vigilance sub-stage combinations (see Supplement Table S1), there was a significant main effect of status for the highest stages 0/A1 (p = 0.004), a significant main effect of status (p = 0.013) and recording time (p = 0.016) for intermediate stages B1 as well as a significant main effect of status (p = 0.015) for lowest stages B2/3 + C. Testing for baseline differences between status groups, the significant main effects of status for stages 0/A1 (F = 4.497; p = 0.044), B1 (F = 6.887; p = 0.015) and B2/3 + C (F = 4.881; p = 0.037) were again seen in the age-adjusted models, in addition there was a significant status * recording time interaction for stages A2/3 (F = 0.966; p = 0.039).

#### Mood and sleepiness ratings

Mood ratings at baseline and after sleep deprivation are shown in Table 4. The rmANCOVA on HDRS_Bech_-scores revealed a highly significant day * status interaction (F = 37.238, p < 0.001). Post-hoc t-tests showed that responders and non-responders did not differed in scores at baseline (t = 1.378, p = 0.180). Changes in subjective mood due to PSD were also found in the ASTS mood ratings, where responders scored significantly lower on sadness and higher on positive mood in the morning after PSD (see Supplement Table S3). Concerning sleepiness ratings in the SSS, the rmANCOVA revealed no main effect of status (F = 0.181, p = 0.675) or day (F = 0.363, p = 0.553) but a trend for a status * day interaction (F = 5.204, p = 0.060). In the ASTS, non-responders scored significantly higher than responders in the morning after PSD concerning tiredness (see Supplement Table S3).

## Discussion

The aim of the current study was to study hypotheses derived from the arousal regulation model of affective disorders^10^, according to which the antidepressant effect of therapeutic sleep deprivation is caused by destabilizing the hyperstable brain arousal regulation typically seen in depressed patients.

When brain arousal regulation (assessed via EEG-vigilance classification using the VIGALL algorithm) was compared between depressed patients and healthy controls before and after partial sleep deprivation (PSD), we found the expected signs of a hyperstable brain arousal regulation in the depressed patients. The Mean Vigilance Value (MVV) of patients compared to healthy controls was higher throughout the 15 min recording independent of the assessment day, showing that lower vigilance stages had less frequently been reached by the patients. Furthermore, within the patient sample, higher Arousal Stability Scores (ASS) were found than in the control group, reflecting delayed declines to lower vigilance stages and thus a more stable brain arousal regulation. It should be noted that our sample was medicated, thus one could assume that the medication may have contributed to the stable arousal regulation pattern. However, our results are in line with previous findings in unmedicated samples^20,21,23^. Besides, the therapeutic effects of antidepressants have been associated with their brain-arousal reducing properties^10,37,38^.

We also found the expected destabilisation of EEG-vigilance after PSD, which resulted in a general increase of lower vigilance stages during the 15 min EEG-recordings at day 2 and thus lower MVV and ASS scores.

However, our hypothesis of more prominent PSD-related destabilisation of EEG-vigilance in patients compared to healthy controls could not be verified. To the contrary, the general reduction in MVV and ASS scores after PSD resulted from pronounced changes in EEG-vigilance within the control sample, whereas the patient group showed only little change in both vigilance parameters after PSD. It can thus be concluded that a destabilisation of brain arousal as a physiological reaction to sleep deprivation occurs in a lesser degree in the presence of a hyperstable brain arousal regulation. It is remarkable that changes in subjective mood ratings were to the opposite, as an improvement in mood was found in the patient sample after PSD. On the other hand, controls reported on higher tiredness consistent with their lower and less stable EEG-vigilance regulation after PSD. This well-known reaction to sleep deprivation^26,39^ was not seen in the patient group. Even after PSD patients remained prominently in higher EEG-vigilance stages and thus still exhibited a stable brain arousal regulation, although they still scored (non-significantly) poorer than controls on subjective tiredness.

As reported above, depression scores were significantly lower in the patient sample after sleep deprivation compared to baseline. According to the arousal model of affective disorders^10^, this reduction in symptom severity should have been facilitated by destabilizing the hyperstable brain arousal regulation. This led to the second hypothesis that sleep deprivation would cause an antidepressant effect especially in those patients showing a hyperstable EEG-vigilance regulation at baseline. Therefore, we divided the patient sample into PSD responders and non-responders. The responder/non-responder ratio was comparable to the response rate typically seen in depressed patients treated with sleep deprivation^1^. Responders and non-responders generally differed concerning EEG-vigilance as the MVV scores of responders were generally lower, confirming a higher prevalence of lower EEG-vigilance stages within the responders. A group difference was also seen in the ASS scores after PSD, with responders scoring lower than non-responders, meaning that they reached lower vigilance stages earlier. Interestingly, the EEG-vigilance of non-responders showed little to no difference between both assessment days whereas responders declined more frequently to lower EEG-vigilance stages after PSD. However, neither a significant main effect of assessment day nor an interaction of status and day was found for MVV or ASS scores. Still, the destabilisation of brain arousal in responders went in line with their decrease in depressive symptoms after sleep deprivation therapy. Interestingly, a similar effect could be shown for pharmacological antidepressant therapy which resulted in reduced EEG-vigilance in responders^38^.

For clinical purposes it would be most relevant if baseline EEG-vigilance measures could be used to predict response to sleep deprivation. A high level of basic activation has been described to be a response predictor to sleep deprivation^40^ and patients who showed higher motor activity during the sleep deprivation intervention have been shown to respond better than those with little activation in the night of sleep deprivation^41^. Therefore, our hypothesis had been that PSD would have an antidepressant effect especially in those patients exhibiting a hyperstable brain arousal regulation. Our results, however, did not support this hypothesis: At baseline there was a significant group difference in mean vigilance values (MVV) between responders and non-responders. Responders were found to have lower MVV scores than non-responders, due to reaching lower vigilance stages more frequently. A direct comparison of the ASS at baseline showed no significant difference between the two patient groups. The ASS scores of responders were again slightly lower compared to the non-responders, attesting to the fact that non-responders remained consistently in higher vigilance stages throughout the EEG recording. It is thus to be concluded that those patients showing a somewhat less stable brain arousal regulation might respond better to sleep deprivation therapy rather than those with a hyperstable one. This seems to stand in contrast to pharmacological antidepressant therapy where responders to antidepressant medication have been shown to exhibit a higher brain arousal than non-responders before medication onset^38^, although opposite results have also been reported based on shorter EEG recordings^42^.

There are several EEG-based measures (e.g. frequency band power, alpha asymmetry, theta cordance and event-related potentials) that have been discussed as potential biomarkers for treatment response in affective disorders^43,44^. Some results might seem contradictory to our results, for example increased alpha has been linked to antidepressant response and is interpreted as being associated with a slightly less aroused state. This is also recognized within the vigilance framework, where the highest vigilance stage 0 is characterized by a non-alpha EEG and alpha activity characterizes A-stages reflecting relaxed wakefulness. However, most EEG-markers are based on the temporo-spatial patterns of EEG activity, which is largely affected by changes in the general level of brain arousal, and they are therefore derived from EEG-recordings performed under strictly vigilance-controlled conditions. Therefore, research on EEG-biomarkers mainly relies on short EEG-recordings, primarily utilizing EEG-epochs from higher vigilance stages. EEG epochs containing stages B1, B23 or C, which are encountered more and more frequently with increasing recording duration, are typically excluded. It is therefore possible that subjects who might be considered less aroused within the first epochs of an EEG-recording could nonetheless exhibit a more stable arousal regulation during prolonged and non-vigilance controlled recordings. The purpose of this paper was to investigate implications of the arousal regulation model and the EEG-recordings corresponded to the requirements of EEG-vigilance research. Therefore, results cannot be readily compared with studies relying on different methodological approaches.

## Limitations

There are certain limitations of the study that should be discussed. One source of uncertainty is that sleep deprivation conditions between the patient and the control sample could not be made fully comparable. Due to administrative reasons, controls could not perform sleep deprivation in the same clinical setting as patients. We instructed the controls to orient themselves on the procedures of the psychiatric ward and monitored both patients and controls using actigraphy. Patients spent the night in a relatively calm environment with limited activity capabilities whereas controls could occupy themselves at their own discretion in their homes. Thus an increased arousal destabilisation in controls may have been triggered by different physical activity and not only by the comparable lack of sleep. One might also question if patients and controls performed the PSD as requested and did not sleep during the following day. Concerning the controls sample, we saw a destabilisation of EEG-vigilance on the day after sleep deprivation, which gives indirect evidence that a PSD was performed. Huang *et al*.^35^ have shown that comparable EEG-vigilance parameters can be expected when healthy subjects are recorded twice under comparable conditions. Such changes in EEG-vigilance were not seen in the patient sample but patients spent the PSD night in a clinical sitting supervised by members of the nursing staff and it can thus be assumed that they actually abided by the protocol. Furthermore, all participants were monitored using actigraphy and those participants for whom signs of non-adherence were found were excluded from the analyses. A second limitation is the relatively small number of patients which resulted in small groups for the responder/non-responder analyses. Further research with larger patient samples is needed to investigate whether a hyperstable EEG-vigilance regulation pattern is a valid predictor for non-response to sleep deprivation as suggested by our data.

## Conclusion

In conclusion, sleep deprivation had a destabilizing effect on brain arousal in healthy controls, whereas depressed patients react heterogeneously. Therefore, on group level patients did not show significant changes in their hyperstable brain arousal regulation. When patients were divided according to their clinical response to sleep deprivation, responders showed some signs of a less stable arousal regulation at baseline and a small decline after sleep deprivation, whereas EEG-vigilance regulation remained relatively unchanged in non-responders. This study ultimately supports general aspects of the arousal model of affective disorders but suggests that a hyperstable arousal regulation is not associated with a positive response to sleep deprivation. More research with higher sampling rates is needed to finally clarify whether or not markers of EEG-vigilance are useful as response predictors.

## Electronic supplementary material

Supplementary Information

## Data Availability

The datasets generated during and/or analysed during the current study are available from the corresponding author on reasonable request.
