# Supplementary material for: Changes in brain arousal (EEG-vigilance) after therapeutic sleep deprivation in depressive patients and healthy controls
Source: Sci Rep. 2018 Oct 10;8:15087. doi: 10.1038/s41598-018-33228-x (PMC6180108; doi:10.1038/s41598-018-33228-x)
Supplement: Supplementary file 1 — Supplementary Information [file 41598_2018_33228_MOESM1_ESM.docx]

**Title:**

Changes in brain arousal (EEG-vigilance) after therapeutic sleep deprivation in depressive patients and healthy controls

**Short title:**

Sleep deprivation and brain arousal changes

**Authors:**

Christian Sander (PhD) ^1^*, Jonathan M. Schmidt^2^*, Roland Mergl (PhD) ^1^, Frank M. Schmidt (MD) ^1^, Ulrich Hegerl (Professor) ^1,2^

* Both authors contributed equally

**Affiliations:**

^1^ Department of Psychiatry and Psychotherapy, University Hospital Leipzig, Germany

^2^ Medical Faculty, University of Leipzig, Germany

**Corresponding author:**

Christian Sander

Department of Psychiatry and Psychotherapy, University Hospital Leipzig,

Semmelweisstrasse 10, D-04103 Leipzig, Germany,

Phone: +49 (0) 341 24558

E-mail: Christian.Sander@medizin.uni-leipzig.de

Supplementary Table S1: Results on changes in amount of EEG-vigilance stages in patients vs. healthy controls (left part) and responders vs. non-responders to sleep deprivation (right part) from baseline to after sleep deprivation

| Patients vs. Controls ^A^ | | Responder vs. Non-Responder ^B^ | |
| --- | --- | --- | --- |
| 0/A1-stages | | 0/A1-stages | |
| ME group | **F = 17.100; p < .001** | ME status | **F = 10.282; p = .004** |
| ME time | **F = 17.191; p < .001** | ME time | F = 1.949; p = .095 |
| ME day | **F = 18.731; p < .001** | ME day | F = 0.396; p = .535 |
| Group * time | F = 1.342; p = .256 | Status * time | F = 1.676; p = .150 |
| Group * day | F = 1.973; p = .168 | Status * day | F = 1.487; p = .235 |
| Time * day | F = 2.066; p = .073 | Time * day | F = 2.057; p = .077 |
| Group * time * day | F = 1.025; p = .403 | Status * time * day | F = 1.016; p = .411 |
| A2/3-stages | | A2/3-stages | |
| ME group | F = 1.961; p =.169 | ME status | F = 0.798; p = .380 |
| ME time | F = 0.800; p =.510 | ME time | F = 0.259; p = .807 |
| ME day | **F = 7.781; p = .008** | ME day | F = 0.259; p = .615 |
| Group * time | F = 0.856; p = .478 | Status * time | F = 0.511; p = .632 |
| Group * day | F = 3.042; p = .089 | Status * day | F = 0.658; p = .425 |
| Time * day | F = 0.531; p = .802 | Time * day | F = 0.774; p = .568 |
| Group * time * day | F = 1.249; p = .278 | Status * time * day | F = 0.624; p = .678 |
| B1-stages | | B1-stages | |
| ME group | **F = 5.108; p = .029** | ME status | **F = 7.275; p = .013** |
| ME time | F = 1.584; p = .169 | ME time | **F = 2.976; p = .016** |
| ME day | F = 0.781; p = .382 | ME day | F = 2.022; p = .168 |
| Group * time | F = 1.827; p = .113 | Status * time | F = 0.744; p = .586 |
| Group * day | F = 0.307; p = .582 | Status * day | F = 0.234; p = .633 |
| **Time * day** | **F = 3.134; p = .015** | Time * day | F = 1.923; p = .120 |
| Group * time * day | F = 0.357; p = .845 | Status * time * day | F = 1.872; p = .128 |
| B2/3+C-stages | | B2/3+C-stages | |
| ME group | **F = 10.700; p = .002** | ME status | **F = 6.841; p = .015** |
| ME time | **F = 18.105; p < .001** | ME time | F = 1.536; p = .207 |
| ME day | **F = 30.180; p < .001** | ME day | F = 0.675; p = .419 |
| Group * time | **F = 4.297; p = .006** | Status * time | F = 1.082; p = .366 |
| Group * day | F = 3.253; p = .079 | Status * day | F = 1.764; p = .197 |
| Time * day | **F = 5.389; p < .001** | Time * day | F = 1.882; p = .138 |
| Group * time * day | F = 1.022; p = .400 | Status * time * day | F = 0.553; p = .655 |

Annotations: ME = Main Effect; Group (depressive patients vs. healthy controls), Status (responders vs. non-responders), time (recording minutes 1-15), day (assessment days: baseline vs. after sleep deprivation).

^A^ repeated measure ANOVA

^B^ repeated measures ANCOVA (with age as covariate)

Supplementary Table S2:

Subjective Mood ratings (ASTS questionnaire) in the morning and at noon in patients vs. healthy controls (left part) at baseline and after partial sleep deprivation (PSD).

| ASTS scale | Day | Time of Day (ToD) | N | Patients  mean (s.d.) | Controls  mean (s.d.) | rmANOVA results | Post hoc:  Patients vs. controls |
| --- | --- | --- | --- | --- | --- | --- | --- |
| Sadness | Baseline | Morning | 24/13 | 13.17 (±4.380) | 4.23 (±2.713) | ME group: F= 37.277; p < .001  ME day: F= 11.946; p = .001  ME ToD: F= 0.674; p = .417  day*group: F= 9.241; p = .004 | t=7.647; p<.001 |
|  |  | Noon |  | 12.00 (±5.082) | 4.15 (±1.519) |  | t=7.008; p<.001 |
|  | After PSD | Morning |  | 9.67 (±4.050) | 4.08 (±2.216) |  | t=5.426; p<.001 |
|  |  | Noon |  | 9.50 (±5.225) | 3.92 (±2.216) |  | t=4.530; p<.001 |
| Hopelessness | Baseline | Morning | 24/13 | 11.46 (±5.556) | 3.46 (±1.127) | ME group: F= 32.064; p < .001  ME day: F= 11.047; p = .002  ME ToD: F= 0.079; p = .780  day*group: F= 9.094; p = .001 | t=6.798; p<.001 |
|  |  | Noon |  | 11.63 (±5.190) | 3.00 (±0.00) |  | t=8.141; p<.001 |
|  | After PSD | Morning |  | 9.17 (±4.949) | 3.23 (±0.599) |  | t=5.798; p<.001 |
|  |  | Noon |  | 9.17 (±5.113) | 3.00 (±0.00) |  | t=5.908; p<.001 |
| Tiredness | Baseline | Morning | 24/13 | 18.58 (±4.845) | 10.54 (±5.939) | ME group: F= 13.691; p = .001  ME day: F= 19.328; p < .001  ME ToD: F= 5.054; p = .031  day*group: F= 13.217; p = .001 | t=4.454; p<.001 |
|  |  | Noon |  | 16.75 (±5.050) | 7.69 (±3.545) |  | t=5.731; p<.001 |
|  | After PSD | Morning |  | 18.88 (±5.788) | 16.15 (±5.080) |  | t=1.422; p=.164 |
|  |  | Noon |  | 17.79 (±6.122) | 16.15 (±6.336) |  | t=0.768; p=.448 |
| Positive Mood | Baseline | Morning | 24/13 | 9.83 (±4.622) | 21.92 (±7.285) | ME group: F= 30.300; p < .001  ME day: F= 0.407; p = .528  ME ToD: F= 23.172; p < .001  day*group: F= 5.770; p = .022 | t=-5.421; p<.001 |
|  |  | Noon |  | 13.63 (±6.940) | 25.08 (±6.994) |  | t=-4.779; p<.001 |
|  | After PSD | Morning |  | 13.38 (±5.609) | 19.92 (±5.171) |  | t=.3.481; p=.001 |
|  |  | Noon |  | 15.92 (±7.661) | 23.69 (±4.803) |  | t=-3.785; p=.001 |
| Anger | Baseline | Morning | 25/13 | 6.28 (±3.385) | 4.23 (±2.713) | ME group: F= 4.215; p = .047  ME day: F= 0.027; p = .871  ME ToD: F= 5.665; p = .023  day*group: F= 0.115; p = .737 | t=2.025; p=.052 |
|  |  | Noon |  | 4.80 (±2.614) | 3.23 (±0.832) |  | t=2.746; p=.010 |
|  | After PSD | Morning |  | 5.36 (±4.102) | 4.00 (±1.472) |  | t=1.151; p=.257 |
|  |  | Noon |  | 5.28 (±3.470) | 3.62 (±1.193) |  | t=2.165; p=.038 |

Annotations: ASTS = “Aktuelle Stimmungsskala” (a German short form of the Profile of Mood States, POMS)

ME = Main Effect; group (depressive patients vs. healthy controls), status (responders vs. non-responders), assessment day (day: baseline vs. after sleep deprivation), and time of day (ToD: morning vs. noon).

Supplementary Table S3:

Subjective Mood ratings (ASTS questionnaire) in the morning and at noon at baseline and after partial sleep deprivation (PSD) in responders versus non-responders to PSD.

| ASTS scale | Day | Time of Day (ToD) | N | Responder  mean (s.d.) | Non-Responder  mean (s.d.) | rmANCOVA results | Post hoc:  Resp. vs. Non-Resp. |
| --- | --- | --- | --- | --- | --- | --- | --- |
| Sadness | Baseline | Morning | 15/9 | 12.73 (±4.574) | 13.89 (±4.197) | ME status: F= 1.208; p = .284  ME day: F= 1.106; p = .305  ME ToD: F= 0.033; p = .858  day*status: F= 8.204; p = .009 | t= -0.617; p= .543 |
|  |  | Noon |  | 12.60 (±4.517) | 11.00 (±6.062) |  | t= 0.739; p= .468 |
|  | After PSD | Morning |  | 8.47 (±3.441) | 11.67 (±4.387) |  | t= -1.991; p= .059 |
|  |  | Noon |  | 8.20 (±4.960) | 11.67 (±5.196) |  | t= -1.629; p= .118 |
| Hopelessness | Baseline | Morning | 15/9 | 11.80 (±5.979) | 10.89 (±5.061) | ME status: F= 0.055; p = .0.816  ME day: F= 1.439; p = .244  ME ToD: F= 0.197; p = .662  day*status: F= 10.823; p = .003 | t= 0.382; p= .706 |
|  |  | Noon |  | 12.60 (±4.968) | 10.00 (±5.431) |  | t= 1.119; p= .243 |
|  | After PSD | Morning |  | 8.80 (±5.102) | 9.78 (±4.919) |  | t= -0.460; p= .650 |
|  |  | Noon |  | 8.33 (±4.938) | 10.56 (±5.388) |  | t= -1.032; p= .313 |
| Tiredness | Baseline | Morning | 15/9 | 17.47 (±4.779) | 20.44 (±4.613) | ME status: F= 2.601; p = .122  ME day: F= 2.835; p = .107  ME ToD: F= 0.289; p = .596  day*status: F= 6.281; p = .020 | t= -1.497; p= .149 |
|  |  | Noon |  | 17.27 (±4.847) | 15.89 (±5.555) |  | t= 0.639; p= .530 |
|  | After PSD | Morning |  | 16.40 (±5.221) | 23.00 (±4.243) |  | t= -3.202; p= .004 |
|  |  | Noon |  | 16.27 (±6.239) | 20.33 (±5.292) |  | t= -1.631; p= .117 |
| Positive Mood | Baseline | Morning | 15/9 | 9.20 (±4.246) | 10.89 (±5.278) | ME status: F= 0.141; p = .711  ME day: F= 0.448; p = .510  ME ToD: F= 3.973; p = .059  day*status: F= 7.094; p = .015 | t= -0.862; p= .398 |
|  |  | Noon |  | 12.93 (±5.351) | 14.78 (±9.271) |  | t= -0.545; p= .596 |
|  | After PSD | Morning |  | 14.87 (±6.128) | 10.89 (±3.689) |  | t= 1.757; p= .093 |
|  |  | Noon |  | 18.07 (±7.497) | 12.33 (±6.874) |  | t= 1.869; p= .075 |
| Anger | Baseline | Morning | 16/9 | 5.56 (±3.054) | 7.56 (±3.745) | ME status: F= 1.293; p = .268  ME day: F= 2.830; p = .107  ME ToD: F= 0.009; p = .927  day*status: F= 0.572; p = .457 | t= -1.445; p= .162 |
|  |  | Noon |  | 4.31 (±2.182) | 5.67 (±3.202) |  | t= -1.258; p= .221 |
|  | After PSD | Morning |  | 4.94 (±4.494) | 6.11 (±3.408) |  | t= -0.679; p= .504 |
|  |  | Noon |  | 4.56 (±2.920) | 6.56 (±4.157) |  | t= -1.406; p= .173 |

Annotations: ASTS = “Aktuelle Stimmungsskala” (a German short form of the Profile of Mood States, POMS)

ME = Main Effect; group (depressive patients vs. healthy controls), status (responders vs. non-responders), assessment day (day: baseline vs. after sleep deprivation), and time of day (ToD: morning vs. noon).
